# A theoretical model of the relationship between the *h*-index and other simple citation indicators

**DOI:** 10.1007/s11192-017-2351-9

**Published:** 2017-03-20

**Authors:** Lucio Bertoli-Barsotti, Tommaso Lando

**Affiliations:** 1 Department of Management, Economics and Quantitative Methods, University of Bergamo, via dei Caniana 2, 24127 Bergamo, Italy; 2 Department of Finance, VŠB -TU Ostrava, Sokolskà 33, 70121 Ostrava, Czech Republic

**Keywords:** Journal ranking, *h*-index for journals, Journal impact factor, Glänzel–Schubert formula, Geometric distribution, Lambert *W* function, 62P99, C46

## Abstract

Of the existing theoretical formulas for the *h*-index, those recently suggested by Burrell (J Informetr 7:774–783, 2013b) and by Bertoli-Barsotti and Lando (J Informetr 9(4):762–776, 2015) have proved very effective in estimating the actual value of the *h*-index Hirsch (Proc Natl Acad Sci USA 102:16569–16572, 2005), at least at the level of the individual scientist. These approaches lead (or may lead) to two slightly different formulas, being based, respectively, on a “standard” and a “shifted” version of the geometric distribution. In this paper, we review the genesis of these two formulas—which we shall call the “basic” and “improved” Lambert-*W* formula for the *h*-index—and compare their effectiveness with that of a number of instances taken from the well-known Glänzel–Schubert class of models for the *h*-index (based, instead, on a Paretian model) by means of an empirical study. All the formulas considered in the comparison are “ready-to-use”, i.e., functions of simple citation indicators such as: the total number of publications; the total number of citations; the total number of cited paper; the number of citations of the most cited paper. The empirical study is based on citation data obtained from two different sets of journals belonging to two different scientific fields: more specifically, 231 journals from the area of “Statistics and Mathematical Methods” and 100 journals from the area of “Economics, Econometrics and Finance”, totaling almost 100,000 and 20,000 publications, respectively. The citation data refer to different publication/citation time windows, different types of “citable” documents, and alternative approaches to the analysis of the citation process (“prospective” and “retrospective”). We conclude that, especially in its improved version, the Lambert-*W* formula for the *h*-index provides a quite robust and effective ready-to-use rule that should be preferred to other known formulas if one’s goal is (simply) to derive a reliable estimate of the *h*-index.

## Introduction

Some simple and basic bibliometric indicators, such as the total number of citations *C*, the total number of publications with at least a number of citations *k* each, *T*_*k*_, the total number of citations for the *t* most cited papers, *C*_*t*_, the average number of citations per paper (ACPP), $m = C/T$ (where, hereafter, *T* stands for *T*_0_), as well as the *h*-index (Hirsch 2005; Braun et al. 2006; Schubert and Glänzel 2007; Harzing and van der Wal 2009), are routinely used to measure the relevance and citation impact of journals when computed according to suitable, pre-specified timeframes. In particular, time-limited versions of the ACPP lead to different types of “impact factors”, with possible variants defined according to different pre-specified publication and citation time windows, and also depending on the degree of overlap between these timeframes (synchronous and diachronous impact factors; Ingwersen et al. 2001). Similarly, alternative versions of the *h*-index have been defined (synchronous and diachronous *h*-indexes; Bar-Ilan 2010). In general, all these indicators merge information about the number of citations received by a journal within a pre-specified time window—typically a huge amount of data—into a single representative value interpretable as a measure of a journal’s “quality”. Their computation requires knowledge of the entire citation pattern, or at least most of it. In recent years, a certain interest has been shown in developing theoretical models with which to “estimate” one such indicator given the values of certain others. Well-known representative examples are theoretical models with which to obtain the value of the *h*-index, *h*:

- as a function of *C* (Hirsch 2005),
- as a function of *T* (Egghe and Rousseau 2006),
- as a function of *T*_1_ (Burrell 2013a),
- as a function of *C* and *T* (Glänzel 2006; Iglesias and Pecharroman 2007; Schubert and Glänzel 2007; Bletsas and Sahalos 2009; Egghe et al. 2009; Egghe and Rousseau 2012),
- as a function of *C*, *T*_1_ and *C*_1_ Bertoli-Barsotti and Lando (2015);

but also theoretical models with which to estimate *C*, as a function of *h* (Petersen et al. 2011), or as a function of *m* and *h* (Egghe et al. 2009), or as a function of *T* and *h* (Burrell 2013b), and so on. These models—usually based, in their turn, on the assumption of a specific probabilistic model for the citation distribution—may be effective, for instance, when the indicator of interest cannot be obtained directly because it is not accessible, or when the availability of citation data is incomplete. For example, there may be the case in which *h* is not available but we know *C* and *T* (Glänzel 2006; Schubert and Glänzel 2007; Bletsas and Sahalos 2009), or the case in which we have to impute missing values of impact factors using the availability of the *h*-index as a predictor (Bertocchi et al. 2015).

In particular, in this paper we focus mainly on the problem of obtaining an explicit “universal” formula for estimating the actual value of the *h*-index. Recently, Burrell (2013b) and Bertoli-Barsotti and Lando (2015) introduced a model that has proved very effective in estimating the actual value of the *h*-index for individual scientists. More precisely, these approaches lead (or may lead) to two slightly different formulas, being based, respectively, on a “standard” and a “shifted” version of the geometric distribution. In the first part of section ‘Methods’ we present a (functional) equation, based on the geometric distribution, that constitutes a theoretical basis for both these approaches. Indeed, this equation allows us to derive a *closed*-*form* estimator of the *h*-index, expressed as a function of (some of) the above citation metrics. We shall call this estimator, for reasons which will be apparent below, the Lambert-*W* formula for the *h*-index.

In the related scientific literature, authors often limit their analysis to the problem of estimating the unknown parameters of a suggested theoretical parametric model for the *h*-index, under the assumption of *knowing* the real values of the *h*-index. Instead, in this paper we consider the more practical (and in a certain sense, opposing) problem of determining the (unknown) *h*-index on the basis of a ready-to-use formula for it. Then, in our empirical analyses we will use the actual values of the *h*-index but only to evaluate, a posteriori, the performance of the proposed ready-to-use formulas and not to determine (maybe for interpretative reasons) unknown parameters of a theoretical parametric model. In this paper, we will concentrate on the case of the *h*-index for journals (Braun et al. 2006). One of the major differences between the cases of an individual scientist and a journal is that, in the latter, the *h*-index should be computed in a “timed” version, i.e. limited to suitable, usually relatively short, publication and citation time windows. In this regard, it should be noted that a familiar definition such as “a journal has index *h* if *h* of its publications each have at least *h* citations and the other publications each have no more than *h* citations” is somewhat inaccurate because it does not specify the time windows to be considered for the calculation of *h*. One of the aims of our study will also be to test the robustness of the formula empirically against different possible choices of (1) length of the time windows and (2) type of approach adopted for analyzing the citation process: “prospective” (diachronous) or “retrospective” (synchronous) (Glänzel 2004). We shall also focus on a comparison of effectiveness between the Lambert-*W* formula for the *h*-index and a popular class of alternative models, related to the so-called Glänzel–Schubert formula, that have already been proved to be highly correlated to the *h*-index.

In the second part of section ‘Methods’ we review the existing literature on the Glänzel–Schubert family of models (and related models) and discuss some problematic aspects linked to the presence of unknown parameters in their expressions. Then, in section ‘Two empirical studies’, we report the results of an empirical comparison between the Lambert-*W* formula for the *h*-index and these alternative models, using two different dataset of journals. For this task, we downloaded citation data from the Scopus database on about 100,000 and 20,000 publications, respectively, for the first and the second dataset. Based on the results of our research study, we conclude that the Lambert-*W* formula for the *h*-index provides an effective ready-to-use rule that should be preferred to other known formulas if one’s goal is (simply) to derive a reliable estimate of the *h*-index.

## Methods

### Models of the relationship between *h* and other simple metrics based on citation counts

#### A basic equation connecting *h*, *T* and *C*

A model of a hypothetical equation of the type

1 $$f\left( {h,T,C} \right) = 0$$

is sought, connecting *h*, *T* and *C*. Naturally, we do not assume a deterministic relationship among observed values of *h*, *T* and *C*, rather, we shall determine a “probabilistic” relationship. Indeed, the problem addressed here is that of deriving a formula for predictions. In particular, we try to identify a model that is able to predict one input-term given the other two (e.g. *h* given *T* and *C*, or *C* given *h* and *T*, or, which is the same, *C*/*T* given *h* and *T*, and so on). A preliminary solution of the functional Eq. (1) can be obtained by “assuming” (which here represents a simple working hypothesis) the *geometric distribution* (GD) with parameter *P*,

2 $$p\left( x \right) = \frac{{P^{x} }}{{\left( {1 + P} \right)^{x + 1} }},\quad x = 0,1,2, \ldots ,$$

where *p*(*x*) gives the probability of observing *x* and *P*, *P* > 0, represents the expectation of the GD (Johnson et al. 2005, p. 210). Then the value $n\left( x \right) = Tp\left( x \right)$ expresses the “expected” number of articles with *x* citations (size-frequency function). Now, since for every *k*, $k \in \left\{ {1,2,3, \ldots } \right\}$, $\sum\nolimits_{x = 0}^{k - 1} {p(x)} = 1 - \left( \frac{P}{1+P}\right)^{k}$, the predicted number of papers with at least *k* citations is

3 $$T_{k} = T \cdot \left( {\frac{P}{1 + P}} \right)^{k} .$$

By definition of the *h*-index, *h*, this yields the equation $\left( {\frac{P}{1 + P}} \right)^{h} - \frac{h}{T} = 0$. Then, assuming $m = C/T$ as an estimate of the expectation *P* (see Johnson et al. 2005, Eq. 5.12, p. 211), we derive the following model of functional equation

4 $$\left( {\frac{m}{1 + m}} \right)^{h} - \frac{h}{T} = 0.$$

We note in passing that this model yields, as a byproduct, the formula $n\left( 0 \right)/{T} = \left( {1 + m} \right)^{ - 1}$ for the “uncitedness factor”, providing proof of the result conjectured by Hsu and Huang (2012) (see also Egghe 2013; Burrell 2013c). This equation represents a theoretical model of the relationship among the *h*-index, the number of publications *T* and the ACPP, *m*. Equation (4) can be solved with respect to any of its arguments. In particular,

- Given *h* and *T*, we easily obtain an estimate $P^{*}$ of the expectation *P* as follows:
  5 $$P^{*} = \frac{{\left( {\frac{h}{T}} \right)^{1/h} }}{{1 - \left( {\frac{h}{T}} \right)^{1/h} }},$$
  and
- Given *T* and *C*, we obtain an estimate of *h* as follows. Equation (4) is equivalent to $sa^{s} = - T$, where $a = \frac{m}{1 + m}$ and $s = - h$. Then, multiplying each side of the latter equation by log *a*, and substituting $z = s\log a$, we obtain $z e^{z} = - T\log a$, which leads immediately to the solution
  6 $$z = W\left( { - T\log a} \right),$$
  where $W\left( \cdot \right)$ represents the so-called *Lambert*-*W* function (Corless and Jeffrey 2015). Remember that the *Lambert*-*W* function is the function *W*(*y*) satisfying $y = W\left( y \right) e^{W\left( y \right)}$, and can be currently computed using mathematical software, for example the Mathematica^®^ 10.0 software package (Wolfram Research, Inc. 2014; it is implemented in the Wolfram Language as “LambertW”), or also using the R statistical computing environment (R Development Core Team 2012).
  Hence
  7 $$- h\log \frac{m}{1 + m} = W\left( { - T\log \frac{m}{1 + m}} \right),$$
  that is, equivalently,
  8 $$h_{W}^{\left( 0 \right)} = \frac{{W\left( {T\log \left( {1 + m^{ - 1} } \right)} \right)}}{{\log \left( {1 + m^{ - 1} } \right)}},$$
  where we have adopted a new symbol for differentiating the “predicted” *h*-index, $h_{W}^{\left( 0 \right)}$, from the actual value *h* of the *h*-index. Note that the GD approach has been previously suggested by Burrell (2007, 2013b, 2014) but without giving an explicit formula, in closed form, for the estimation of the *h*-index.

#### An equation connecting *h*, *T*_1_ and *C*

As a general rule, one should expect that knowledge of other (i.e., other than *m* and *T*) simple summary statistics of the raw citation data will help increase the precision of the *h*-index estimate. Indeed, if we also assume that we know *T*_1_, a modified version of the above formulas can be easily introduced by taking the *shifted*-*geometric distribution* (SGD) with parameter Q

9 $$p\left( y \right) = \frac{{\left( {Q - 1} \right)^{y - 1} }}{{Q^{y} }},\quad y = 1,2, \ldots ,$$

where *p*(*y*) represents the probability of observing the number of citations *y* of a paper cited at least once, and *Q*, *Q* > 1, represents the expectation of the SGD. Since for every *k*, $k \in \left\{ {1,2,3, \ldots } \right\}$, $\sum\nolimits_{y = 1}^{k} {p\left( y \right)} = 1 - \left( {\frac{Q - 1}{Q}} \right)^{k}$, then $T_{1} \left( {\frac{Q - 1}{Q}} \right)^{k}$ represents the number of papers with at least *k* + 1 citations. Then, assuming $m_{1} = {C \mathord{\left/ {\vphantom {C {T_{1} }}} \right. \kern-0pt} {T_{1} }}$, the average number of citations of articles that have been cited at least once, as a proxy for the expectation Q, we derive the following functional equation

10 $$\left( {\frac{{m_{1} - 1}}{{m_{1} }}} \right)^{h - 1} - \frac{h}{{T_{1} }} = 0.$$

This equation can be solved with respect to any of its arguments. In particular,

(c) Given *h* and *T*_1_, we obtain
  11 $$Q^{*} = \left( {1 - \left( {\frac{h}{{T_{1} }}} \right)^{{{1 \mathord{\left/ {\vphantom {1 {\left( {h - 1} \right)}}} \right. \kern-0pt} {\left( {h - 1} \right)}}}} } \right)^{ - 1}$$
  and
(d) Given *T*_1_ and *C*, and following a completely analogous sequence of steps as in the above point (b), we obtain the estimate of *h*
  12 $$h_{W}^{\left( 1 \right)} = \frac{ - 1}{{\log \left( {1 - m_{1}^{ - 1} } \right)}} \cdot W\left( {\frac{{T_{1} }}{{1 - m_{1}^{ - 1} }} \cdot \log \left( {1 - m_{1}^{ - 1} } \right)} \right).$$

#### A formula for the *h*-index, as a function of *T*_1_, *C* and *C*_1_

If we also know the total number of citations of the most cited paper, *C*_1_, we can hope to improve the accuracy of the above formula $h_{W}^{\left( 1 \right)}$ further. Indeed, with the use of the trimmed mean—that is, the sample mean obtained omitting the most highly cited paper—$\tilde{m}_{1} = {{\left( {C - C_{1} } \right)} \mathord{\left/ {\vphantom {{\left( {C - C_{1} } \right)} {\left( {T_{1} - 1} \right)}}} \right. \kern-0pt} {\left( {T_{1} - 1} \right)}}$ instead of *m*_1_, we obtain a modified (improved) version of the above formula, which we shall define $\tilde{h}_{W}^{\left( 1 \right)}$,

13 $$\tilde{h}_{W}^{\left( 1 \right)} = \frac{ - 1}{{\log \left( {1 - \tilde{m}_{1}^{ - 1} } \right)}} \cdot W\left( {\frac{{T_{1} }}{{1 - \tilde{m}_{1}^{ - 1} }} \cdot \log \left( {1 - \tilde{m}_{1}^{ - 1} } \right)} \right).$$

As is well known, citation distributions are highly skewed; hence the sample mean is distorted by extreme values. In particular, the presence of individual highly-cited papers tends to overestimate *C*, and consequently $h_{W}^{\left( 1 \right)}$, in comparison to the true *h*-index—that is clearly insensitive to a single very highly cited paper. In this sense, the use of a trimmed mean is simply a technique for reducing this possible bias.

To summarize, we have: $h_{W}^{\left( 0 \right)} = h_{W}^{\left( 0 \right)} \left( {C,T} \right)$ or also, equivalently, $h_{W}^{\left( 0 \right)} = h_{W}^{\left( 0 \right)} \left( {T,m} \right)$, and $\tilde{h}_{W}^{\left( 1 \right)} = \tilde{h}_{W}^{\left( 1 \right)} \left( {C,C_{1} ,T_{1} } \right)$ or also, equivalently, $\tilde{h}_{W}^{\left( 1 \right)} = \tilde{h}_{W}^{\left( 1 \right)} \left( {T_{1} ,\tilde{m}_{1} } \right)$. We shall refer to these formulas as Lambert-*W* formulas for the *h*-index, respectively, in a “basic”, $h_{W}^{\left( 0 \right)}$, and an “improved” version, $\tilde{h}_{W}^{\left( 1 \right)}$. The formula $\tilde{h}_{W}^{\left( 1 \right)}$ has been considered elsewhere Bertoli-Barsotti and Lando (2015) for the estimation of the *h*-index for individual scientists.

### Theoretical parametric models for the *h*-index related to the Glänzel–Schubert formula

A well-known alternative “theoretical model of the dependence of the citation *h*-index on the sample size and the sample’s mean citation rate” (Schubert et al. 2009) is the one proposed by Schubert and Glänzel (2007), who noted that the *h*-index is approximately proportional to “a power function of the sample size and the sample mean”, namely to the function $m^{\eta } T^{1 - \eta }$ (Schubert et al. 2009; see also Glänzel 2007, 2008). In applications, this fact has given rise to a plethora of “variants”, as possible parametric models for the *h*-index. It is useful to distinguish each of them with the following nine cases.

- Iglesias and Pecharroman (2007) derived the following one-parameter family of models of the *h*-index:
  14 $$h_{\text{IP}} \left( \eta \right) = \left( {\frac{2\eta - 1}{\eta }} \right)^{\eta } m^{\eta } T^{1 - \eta } ,$$
  where $\eta > 0.5$ (the formula was reported by Iglesias and Pecharroman with parameter ${{\left( {1 - \eta } \right)} \mathord{\left/ {\vphantom {{\left( {1 - \eta } \right)} \eta }} \right. \kern-0pt} \eta }$). Glänzel (2008) estimated this model in an empirical comparative study of *h*-index for journals. He found that the estimate of the power parameter depends on the length of the citation window considered. In particular, he found that the formula $h_{\text{IP}} \left( {2/3} \right)$ (*α* = 2 in his notation, which corresponds to *η* = 2/3 in ours) is appropriate “for small windows comprising an initial period of about 3 years after publication”.
- From the above model, Iglesias and Pecharroman (2007) also obtained, for *η* = 2/3, the ready-to-use formula:
  15 $$h_{\text{IP}} \left( {2/3} \right) = 4^{ - 1/3} m^{2/3} T^{1/3}$$
  (see also Panaretos and Malesios 2009; Vinkler 2009, 2013; Ionescu and Chopard 2013).
- By starting from a continuous probability distribution—a *Pareto distribution of the second kind,* $P\left( {II} \right)\left( {\sigma ,\theta } \right)$ (Johnson et al. 1994, p. 575; Arnold 1983, p. 44), also known as the *Lomax distribution* (Lomax 1954), where $\sigma^{\theta } \left( {\sigma + x} \right)^{ - \theta } ,\;\theta > 0,\;\sigma > 0$, represents the probability of observing a number greater than *x*, *x* > 0—and estimating its expectation $\sigma \left( {\theta - 1} \right)^{ - 1}$ (that exists if $\theta > 1$) by the sample mean *m*, Schubert and Glänzel (2007) (see also Glänzel 2006) derived a slightly more general two-parameter model:
  16 $$h_{\text{G}} \left( {\eta ,\gamma } \right) = \gamma m^{\eta } T^{1 - \eta }$$
  here defined as also reported by Bletsas and Sahalos (2009); see their Eq. (4)), as an approximate (and generalized) solution of the equation
  17 $$Tm^{\theta } \left( {\theta - 1} \right)^{\theta } \left( {\sigma + h} \right)^{ - \theta } = h,$$
  where $\theta = \eta \left( {1 - \eta } \right)^{ - 1}$. In words, model (16) states that “the *h*-index can be approximated by a power function of the sample size and the sample mean” (Schubert et al. 2009). It is important to note that the model $h_{G} \left( {\eta ,\gamma } \right)$ is similar to but different from the above model $h_{\text{IP}} \left( \eta \right)$, because in the former the proportionality constant is not merely a function of the power parameter *η*, while in the latter *γ* represents a *free* parameter. This gives rise to a more flexible model. Malesios (2015) estimated the parameters of model (16) in a study on 134 journals in the field of ecology and 54 journals in the field of forestry sciences. He obtained the best fit, respectively, with the estimates (0.64, 0.7) and (0.66, 0.78) for the pair (*η*, *γ*) (in our parameterization).
- The above Pareto distribution of the second kind $P\left( {II} \right)\left( {\sigma ,\theta } \right)$ has also recently become known as the *Tsallis distribution* (Tsallis and de Albuquerque 2000). More specifically, with reparameterization $\theta = \left( {q - 1} \right)^{ - 1}$ and $\sigma = \left( {q - 1} \right)^{ - 1} \lambda^{ - 1} ,\;q > 1,\;\lambda > 0$, the probability of observing a number greater than *x*, *x* > 0, becomes equal to $\left( {1 + \lambda \left( {q - 1} \right)x} \right)^{{ - \frac{1}{q - 1}}}$ (see Bletsas and Sahalos 2009; Shalizi 2007). Bletsas and Sahalos (2009) suggest obtaining an estimate of the *h*-index as the *numerical* solution of the Eq. (17), that is
  18 $$T\left( {m\frac{2 - q}{q - 1}} \right)^{{\frac{1}{q - 1}}} \left( {m\frac{2 - q}{q - 1} + h} \right)^{{\frac{1}{1 - q}}} = h,$$
  for a pre-specified fixed value of the unknown parameter *q*. Let us call $h_{\text{BS}} = h_{\text{BS}} \left( q \right)$ the (implicit) solution of Eq. (18). It is important to stress that, unlike all the other estimators of *h*-index considered in the present study, a closed-form expression for *h*_T_ does not exist. Nevertheless, in an empirical application to a set of electrical engineering journals, Bletsas and Sahalos (2009) found a very good fit between measured and estimated values of the *h*-index, assuming Tsallis distribution with parameter *q* = 1.5 and *q* = 1.6. It is interesting to note that these values correspond, respectively, to *η* = 2/3 and *η* = 0.625, since $\eta = q^{ - 1}$.
- For a special choice of the power parameter (*η* = 2/3 in the present parameterization) in model (16), Schubert and Glänzel (2007) derived the celebrated one-parameter model
  19 $$h_{\text{SG}} \left( \gamma \right) = \gamma C^{2/3} T^{ - 1/3} = \gamma m^{2/3} T^{1/3} ,$$
  also known as the *Glänzel*–*Schubert model* of the *h*-index. This model has been widely used (mainly for interpretative purposes—i.e. to provide a better understanding of the “mathematical properties” of the *h*-index) because several empirical studies suggest the existence of a strong correlation between *h*-index and $m^{2/3} T^{1/3}$. Its drawback (as with model (16)) is obviously that the value of the proportionality constant *γ* is unknown. Certainly, this parameter can be determined (ex post) empirically, but it is likely to vary from case to case (Prathap 2010a; Alguliev et al. 2014). Then, as a ready-to-use formula for estimating the *h*-index a priori, the Glänzel–Schubert model is in fact unusable. Sometimes researchers find an *ex post* least square estimate of the parameter *γ*, starting from known values of the *h*-index. In different contexts, and for different datasets, the estimate of the *γ* parameter has been found to vary appreciably, in that it turns out to range approximately from 0.7 to 0.95. Indeed, for example, Schubert and Glänzel (2007) found, for *γ*, the estimates 0.73 and 0.76, in a study on the *h*-index for journals, for two different sets of journals, while Csajbók et al. (2007) found an estimate of *γ* of 0.93 in a macro-level analysis of the *h*-index for countries. Instead, other authors, among them Annibaldi et al. (2010), Bouabid et al. (2011) and Zhao et al. (2014), have found values of around 0.8. In quite different contexts (partnership ability and *h*-index for networks) Schubert (2012) and Schubert et al. (2009) have estimated the parameter *γ* of the model $h_{\text{SG}} \left( \gamma \right)$, obtaining values within the range 0.6–0.96.
- In the absence of a specific value of the proportionality constant *γ*, researchers sometimes decide to set *γ* equal to a *fixed* arbitrary value *γ*_0_, obtaining a ready-to-use formula
  20 $$h_{\text{SG}} \left( {\gamma_{0} } \right) = \gamma_{0} m^{2/3} T^{1/3} .$$
  In the framework of the analysis of the *h*-index for journals, ready-to-use formulas for estimating the *h*-index with the formula $h_{\text{SG}} \left( {\gamma_{0} } \right)$ have been adopted, for example, by Bletsas and Sahalos (2009), with the choice $\gamma_{0} = 0.75$. Instead, for example, Ye (2009, 2010) and Elango et al. (2013) adopted the rule to set $\gamma_{0} = 0.9$ for journals and $\gamma_{0} = 1$ for other sources. Abbas (2012) and Vinkler (2013) also adopted the choice $\gamma_{0} = 1$. It is worth noting that the latter value leads to the formula $h_{\text{SG}} \left( 1 \right)$, which coincides with the so-called *p*-index defined by Prathap (2010b). Finally, note that $h_{\text{SG}} \left( {4^{ - 1/3} } \right) = h_{\text{IP}} \left( {2/3} \right)$.
- As noted above, empirical analyses suggest a “strong linear correlation” between the *h*-index and the function $m^{\eta } T^{1 - \eta }$ (Schubert and Glänzel 2007; Glänzel 2007; Schreiber et al. 2012; Malesios 2015). Strictly speaking, this only means that when *h* is plotted against $m^{\eta } T^{1 - \eta }$, the data fall fairly close to a straight line. In other terms, *h* is approximately equal to $\delta + \gamma m^{\eta } T^{1 - \eta }$, for suitable choices of the parameters *δ* and *γ*. Indeed, the following three-parameter model has been considered in literature (see Bador and Lafouge 2010)
  21 $$h_{\text{BL}} \left( {\delta ,\gamma ,\eta } \right) = \delta + \gamma m^{\eta } T^{1 - \eta } .$$
  In a comparative analysis of two samples of 50 journals (taken from the ‘‘Pharmacology and Pharmacy’’ and ‘‘Psychiatry’’ sections of the Journal Citation Reports 2006), Bador and Lafouge (2010) obtained the LS estimates of the parameters *δ* and *γ* for different fixed values of the power parameter *η* (values of “*α* close to 2”, in their parameterization, where $\eta = {\alpha \mathord{\left/ {\vphantom {\alpha {\left( {\alpha + 1} \right)}}} \right. \kern-0pt} {\left( {\alpha + 1} \right)}}$). Their best estimates of the proportionality constant *γ* ranged from 0.7 to 0.8, with an intercept point always very close to 1. Based on these results, $h_{\text{BS}} \left( {\eta ,\gamma } \right)$ and *a fortiori* $h_{\text{SG}} \left( \gamma \right)$, underestimate the *h*-index.
- For the particular choice of the power parameter *η* = 2/3 in the above model $h_{\text{BL}} \left( {\delta ,\gamma ,\eta } \right)$, we obtain the two-parameter model
  22 $$h_{\text{TAB}} \left( {\delta ,\gamma } \right) = \delta + \gamma \cdot m^{2/3} T^{1/3} .$$
  This model directly generalizes the above Glänzel–Schubert model $h_{\text{SG}} \left( \gamma \right)$ by introducing a free intercept parameter, *δ*. Tahira et al. (2013) tested this model in a scientometric analysis of engineering in Malaysian universities. They found the estimates *δ* = −0.28 and *γ* = 0.97.
- Finally, by assuming a linear dependence between the *h*-index and the function $m^{\eta } T^{1 - \eta }$ in a double logarithmic axis plot (log–log plot), one may define the following three-parameter model (see Radicchi and Castellano 2013)
  23 $$h_{RC} \left( {{\varrho },\varphi ,\eta } \right) = {\varrho }\left( {m^{\eta } T^{1 - \eta } } \right)^{\varphi } .$$
  Indeed, after taking logs, this corresponds to a regression relationship between log *h* and the linear model $\xi + \varphi \cdot \log \left( {m^{\eta } T^{1 - \eta } } \right)$, where ${\varrho } = e^{\xi }$. Needless to say, model $h_{\text{RC}}$ is similar to but essentially different from the above models (a)–(h). Radicchi and Castellano (2013) analyzed the scientific profile of more than 30,000 researchers. They found a good linear correlation, in a log–log plot, between the true *h*-index and the values given by the model $h_{\text{RC}} \left( {{\varrho },\varphi ,\eta } \right)$. Using this relationship, they obtained, in particular, the least square estimate of the parameter *η*: $\hat{\eta } = 0.41$. It is quite puzzling to observe that the solution reached by Radicchi and Castellano is out of the parameter space of all the above models (*η* > 0.5).

## Two empirical studies

### A first dataset of journals

#### Journal selection

The Research Evaluation Exercise for the period 2011–2014 named “Valutazione della Qualità della Ricerca 2011–2014” (hereinafter VQR) is a national research assessment exercise organized under the aegis of the Italian Ministry of Education, University and Research for evaluating and ranking all Italian scientific institutions (typically, all national universities and research centers), on the basis of the quality of their research outcomes. The results obtained are particularly important because they determine the allocation of government funding to Italian universities. The VQR is carried out under the responsibility of a National Agency for the Evaluation of University and Research, the “Agenzia Nazionale di Valutazione del Sistema Universitario e della Ricerca” (ANVUR), and is organized with reference to 14 different academic fields, or Areas. The research assessment is actually conducted by Groups of Evaluation Experts (GEV, in the Italian acronym), one for each Area. For our first empirical analysis, we consider the so-called Area 13—Scienze economiche e statistiche—Economics and Statistics. The evaluation of each researcher is based on the quality of his/her research outcomes published during the period 2011–2014. As a general rule, the evaluation of a research product for Area 13 is made at journal-level. This means that journal bibliometric indicators are used as surrogate measures to quantify the quality of each individual research product (published in that journal). For this purpose, a list of “relevant” journals for Area 13 has been compiled by the corresponding GEV (the so-called GEV 13) and suitable journal-based metrics are extracted to this end from three sources, that is: Web of Science (WoS), Scopus, and Google Scholar (GS). The full list of the “relevant” journals for Area 13 includes 2717 journals and may be found on the ANVUR website (www.anvur.org). Each journal on the Area 13 list was individually assigned to one of five sub-areas, among them “Statistics and Mathematical Methods” (S&MM). For the purpose of our case study, we selected a somewhat homogeneous list of journals using the following steps:

- we considered all and only the journals (568 journals) belonging to the sub-area S&MM;
- to facilitate possible comparisons between databases, the journals selected were subsequently restricted to only those (253) journals indexed by all three databases: WoS, Scopus and GS;
- we excluded 15 journals with incomplete issues within the period under investigation, 2010–2014;
- finally, in order to preserve the homogeneity of the sample, we excluded 6 journals with a “too large” number of published papers (more than 2000) and 1 journal that publishes only online.

Our final sample included 231 journals. According to the Scopus classification, these journals belong to a number of different “Subject Areas”. Table 1 shows the “Subject Areas” in which the 231 journals selected from the S&MM list are placed by Scopus (it should be recalled that Scopus classifies journal titles into 27 major thematic categories and a journal may belong to more than one category).

**Table 1:** Scopus “Subject Areas” of the 231 journals within the S&MM list

Subject area	Count	%
Mathematics	239	38.3
Decision sciences	79	12.7
Computer science	63	10.1
Social sciences	51	8.2
Engineering	45	7.2
Economics, econometrics and finance	37	5.9
Medicine	23	3.7
Business, management and accounting	17	2.7
Environmental science	13	2.1
Others	57	9.1

#### Estimating the *h*-index

After selecting the S&MM list of journals, we retrieved citation data from the Scopus database. According to the VQR time-span, we considered all documents within the publication window of 5 years (2010–2014) (in fact GEV13 considers the 5-year Google Scholar’s *h*-index, for the period 2010–2014) and the citations that these items received until the time of accessing the database (last week of December 2015). This means a 6-year citation window, 2010–2015, over a 5-year publication window: 2010–2014. Harzing and van der Wal (2009) considered similar timeframes in a study on a set of journals in the area of economics and business. Overall, the dataset obtained included 99,409 publications receiving (until December 2015) a total of 485,628 citations. The complete list of the 231 journals in the S&MM dataset is reported in Table 2, where each journal is identified by its ISSN code. For each journal, we manually computed, on the basis of the citations downloaded, the actual value *h* of the *h*-index, as: the largest number of papers published in the journal between 2010 and 2014 and which obtained at least *h* citations each, from the time of publication until December 2015. Table 2 reports, for each journal, the *h*-index, *h*, and its estimates, obtained (1) with the Lambert-*W* formulas for the *h*-index, $h_{W}^{\left( 0 \right)}$, $\tilde{h}_{W}^{\left( 1 \right)}$, and, as a comparison, (2) with the Glänzel–Schubert formula, $h_{\text{SG}} \left( {\gamma_{0} } \right)$, for different values of the proportionality constant *γ*_0_, namely, 0.63, 0.7, 0.8, 0.9, 1 (note that $\gamma_{0} = 0.63 = 4^{ - 1/3}$ identifies formula $h_{\text{IP}} \left( {2/3} \right)$), and (3) by means of a numerical solution $h_{\text{BS}} \left( {q_{0} } \right)$ of Eq. (18), for different values of *q*_0_, namely, 1.2, 1.4, 1.6. Table 2 also reports: the total number of citations, *C*; the total number of publications, *T*; the total number of publications cited at least once, *T*_1_; the total number of citations of the most cited paper, *C*_1_. To facilitate comparisons, $h_{W}^{\left( 0 \right)} ,\;\tilde{h}_{W}^{\left( 1 \right)} ,\;h_{\text{SG}} \left( {\gamma_{0} } \right),\;{\text{and}}\;h_{\text{BS}} \left( {q_{0} } \right)$ have all been rounded to the nearest integer to produce numbers in the same range of values as the *h*-index.

**Table 2:** Basic statistics for the S&MM list of journals and the approximations of the Hirsch *h*-index calculated by means of different formulas (rounded values)

#	ISSN code	*C*	*T*	*T*_1_	$C_{\text{1}}$	*h*	$h_{W}^{\left( 0 \right)}$	$\tilde{h}_{W}^{\left( 1 \right)}$	*h*_SG_ (.63)	*h*_SG_ (.7)	*h*_SG_ (.8)	*h*_SG_ (.9)	*h*_SG_ (1)	$h_{\text{BS}} \;\left( {1.2} \right)$	$h_{\text{BS}} \;\left( {1.4} \right)$	$h_{\text{BS}} \;\left( {1.6} \right)$
1	1405-7425	42	152	24	6	3	3	3	1	2	2	2	2	2	2	2
2	1012-9367	276	360	111	14	6	5	6	4	4	5	5	6	4	5	6
3	0017-095X	158	166	71	13	5	5	5	3	4	4	5	5	4	5	5
4	0315-3681	557	427	177	44	9	7	8	6	6	7	8	9	7	8	8
5	1081-1826	201	140	77	12	6	6	6	4	5	5	6	7	5	6	6
6	0957-3720	323	228	122	15	7	7	7	5	5	6	7	8	6	7	7
7	0002-9890	589	351	171	87	9	8	8	6	7	8	9	10	8	9	9
8	0361-0926	2033	1555	754	28	11	9	10	9	10	11	12	14	9	12	14
9	0117-1968	163	120	61	20	5	6	5	4	4	5	5	6	5	5	5
10	1210-0552	405	205	119	31	9	8	8	6	6	7	8	9	7	8	8
11	1056-2176	290	222	101	22	7	6	7	5	5	6	7	7	6	6	6
12	0165-4896	583	320	198	16	10	8	8	6	7	8	9	10	8	9	9
13	0315-5986	166	83	48	24	6	6	6	4	5	6	6	7	6	6	5
14	0736-2994	577	283	176	19	9	9	9	7	7	8	10	11	8	9	9
15	0399-0559	153	86	47	32	5	6	5	4	5	5	6	6	5	5	5
16	1303-5010	658	334	154	56	11	9	10	7	8	9	10	11	9	9	10
17	0927-7099	463	296	162	16	8	7	8	6	6	7	8	9	7	8	8
18	1351-1610	313	150	92	23	8	8	8	5	6	7	8	9	7	7	7
19	1292-8100	191	78	52	22	7	7	7	5	5	6	7	8	6	6	6
20	0361-0918	1036	635	369	45	9	9	9	8	8	10	11	12	9	10	11
21	0269-9648	263	172	84	16	7	7	7	5	5	6	7	7	6	6	6
22	1532-6349	308	141	93	15	7	8	8	6	6	7	8	9	7	7	7
23	0217-5959	522	261	155	33	9	8	9	6	7	8	9	10	8	9	9
24	1018-5895	424	189	115	25	9	8	9	6	7	8	9	10	8	8	8
25	0266-4763	2164	901	518	323	13	12	12	11	12	14	16	17	13	15	16
26	1471-678X	336	138	92	23	8	8	8	6	7	7	8	9	8	8	8
27	0304-4068	737	433	265	25	9	9	8	7	8	9	10	11	8	9	10
28	0020-7276	480	265	158	13	8	8	8	6	7	8	9	10	8	8	8
29	0023-5954	813	337	208	36	11	10	11	8	9	10	11	13	10	11	11
30	1220-1766	526	193	137	31	10	10	9	7	8	9	10	11	9	10	9
31	1226-3192	457	271	137	20	10	8	8	6	6	7	8	9	7	8	8
32	1618-2510	305	172	90	31	8	7	7	5	6	7	7	8	7	7	7
33	1083-589X	739	353	209	20	10	9	10	7	8	9	10	12	9	10	10
34	1048-5252	643	283	189	17	10	9	9	7	8	9	10	11	9	10	10
35	1004-3756	443	140	96	27	9	10	10	7	8	9	10	11	9	9	9
36	1009-6124	979	466	240	56	12	10	11	8	9	10	11	13	10	11	12
37	1120-9763	434	492	165	18	8	6	7	5	5	6	7	7	5	6	7
38	1369-1473	282	140	76	24	8	7	8	5	6	7	7	8	7	7	7
39	1230-1612	346	128	84	32	8	9	8	6	7	8	9	10	8	8	8
40	0026-1335	544	283	171	24	10	8	9	6	7	8	9	10	8	9	9
41	0218-348X	476	167	129	30	9	10	9	7	8	9	10	11	9	9	9
42	0167-7152	3169	1546	945	40	16	12	13	12	13	15	17	19	13	16	18
43	0032-4663	154	103	58	13	6	6	6	4	4	5	6	6	5	5	5
44	0282-423X	405	196	116	20	9	8	8	6	7	8	8	9	8	8	8
45	1748-670X	1933	822	543	36	14	12	12	10	12	13	15	17	12	14	15
46	0094-9655	1649	695	425	55	14	12	12	10	11	13	14	16	12	14	15
47	0039-0402	365	129	86	34	9	9	9	6	7	8	9	10	8	8	8
48	0894-9840	615	331	184	29	9	9	9	7	7	8	9	10	8	9	9
49	0398-7620	679	303	170	66	10	9	10	7	8	9	10	12	9	10	10
50	0219-0257	336	159	102	31	7	8	7	6	6	7	8	9	7	8	7
51	0319-5724	511	206	129	36	10	9	9	7	8	9	10	11	9	9	9
52	0020-3157	772	285	189	60	11	11	10	8	9	10	12	13	10	11	11
53	0898-2112	597	228	149	26	11	10	10	7	8	9	10	12	9	10	10
54	1524-1904	669	301	155	42	12	9	11	7	8	9	10	11	9	10	10
55	0963-5483	719	272	179	24	11	10	11	8	9	10	11	12	10	11	11
56	1547-5816	770	290	201	37	11	10	10	8	9	10	11	13	10	11	11
57	0001-8678	821	269	201	37	11	11	11	9	10	11	12	14	11	12	11
58	0021-9002	1168	477	321	35	13	11	11	9	10	11	13	14	11	12	13
59	0257-0130	719	260	179	18	11	10	11	8	9	10	11	13	10	11	11
60	1026-0226	2306	1036	610	34	15	12	13	11	12	14	16	17	13	15	16
61	0378-3758	3899	1334	907	71	18	15	16	14	16	18	20	23	16	19	21
62	0377-7332	1353	597	348	38	15	11	12	9	10	12	13	15	11	13	13
63	1560-3547	735	249	182	25	11	11	11	8	9	10	12	13	10	11	11
64	0893-4983	793	297	200	36	12	11	11	8	9	10	12	13	10	11	11
65	1387-5841	645	305	178	26	10	9	10	7	8	9	10	11	9	10	10
66	0167-6377	1702	582	399	33	14	13	13	11	12	14	15	17	13	15	15
67	1747-7778	837	135	93	294	10	15	12	11	12	14	16	17	14	14	13
68	1054-3406	1098	429	277	40	13	11	12	9	10	11	13	14	11	12	13
69	1619-4500	493	125	89	38	12	11	11	8	9	10	11	12	10	10	10
70	0143-9782	761	258	179	31	12	11	11	8	9	10	12	13	11	11	11
71	1432-2994	512	207	146	29	9	9	9	7	8	9	10	11	9	9	9
72	0219-4937	304	178	102	21	7	7	7	5	6	6	7	8	6	7	7
73	0033-5177	1734	878	522	42	14	11	11	9	11	12	14	15	11	13	14
74	1748-006X	779	238	184	31	11	11	11	9	10	11	12	14	11	12	11
75	1381-298X	364	113	82	23	9	9	9	7	7	8	9	11	9	9	8
76	0277-6693	825	217	160	61	14	12	12	9	10	12	13	15	12	12	12
77	1435-246X	735	263	175	43	11	11	11	8	9	10	11	13	10	11	11
78	1572-5286	587	158	114	25	12	11	11	8	9	10	12	13	11	11	10
79	1134-5764	458	246	128	59	8	8	8	6	7	8	9	9	8	8	8
80	0932-5026	829	396	210	26	11	10	11	8	8	10	11	12	9	10	11
81	0926-2601	769	286	196	78	10	10	10	8	9	10	11	13	10	11	11
82	0890-8575	333	119	74	47	8	9	8	6	7	8	9	10	8	8	8
83	0219-5259	803	254	179	32	12	11	11	9	10	11	12	14	11	12	11
84	0515-0361	447	150	89	37	11	10	10	7	8	9	10	11	9	9	9
85	0095-4616	626	192	135	46	11	11	11	8	9	10	11	13	10	11	10
86	0233-1934	1191	490	304	24	13	11	12	9	10	11	13	14	11	12	13
87	0167-5923	663	216	152	38	12	11	11	8	9	10	11	13	10	11	11
88	1469-7688	2100	653	404	77	17	14	16	12	13	15	17	19	15	16	17
89	1083-6489	1321	488	330	32	13	12	12	10	11	12	14	15	12	13	14
90	1392-5113	747	202	138	52	13	12	12	9	10	11	13	14	11	12	11
91	1863-8171	404	118	77	34	10	10	10	7	8	9	10	11	9	9	9
92	1380-7870	379	170	103	39	9	8	8	6	7	8	9	9	8	8	8
93	1862-4472	1866	652	438	32	15	13	14	11	12	14	16	17	13	15	16
94	0219-8762	905	300	185	65	15	11	12	9	10	11	13	14	11	12	12
95	0218-1274	5537	1370	1013	136	26	19	20	18	20	23	25	28	21	24	26
96	0747-4938	649	149	113	54	12	12	12	9	10	11	13	14	12	12	11
97	0020-7985	1280	417	268	28	16	12	13	10	11	13	14	16	12	14	14
98	0047-259X	3329	915	650	89	21	17	17	14	16	18	21	23	18	20	21
99	0303-6898	868	256	188	31	12	12	12	9	10	11	13	14	12	12	12
100	1471-082X	405	134	88	35	9	9	9	7	7	9	10	11	9	9	9
101	0924-6703	413	117	79	38	9	10	10	7	8	9	10	11	9	9	9
102	0346-1238	337	128	79	28	9	8	9	6	7	8	9	10	8	8	8
103	0748-8017	2076	534	380	31	19	15	16	13	14	16	18	20	16	17	18
104	1389-4420	793	184	124	124	15	13	12	9	11	12	14	15	12	12	12
105	0146-6216	737	215	155	30	12	11	12	9	10	11	12	14	11	11	11
106	0160-5682	3870	853	663	90	21	19	19	16	18	21	23	26	20	22	23
107	0960-0779	2712	570	443	118	20	18	18	15	16	19	21	23	19	20	20
108	0246-0203	1019	266	206	33	14	13	13	10	11	13	14	16	13	13	13
109	0306-7734	563	147	83	101	12	11	11	8	9	10	12	13	11	11	10
110	1350-7265	1499	375	294	40	15	15	14	11	13	15	16	18	15	15	15
111	0021-9320	910	274	207	22	12	12	12	9	10	12	13	14	12	12	12
112	0218-4885	1036	297	202	81	13	13	13	10	11	12	14	15	12	13	13
113	1945-497X	885	162	130	57	15	14	14	11	12	14	15	17	14	14	13
114	1352-8505	564	192	130	64	10	10	10	7	8	9	11	12	10	10	10
115	0003-1305	670	241	133	43	13	10	11	8	9	10	11	12	10	10	10
116	1076-2787	900	224	163	49	14	13	13	10	11	12	14	15	13	13	12
117	1862-5347	524	125	79	63	11	11	11	8	9	10	12	13	11	11	10
118	0022-4715	5302	1246	966	91	24	20	20	18	20	23	25	28	21	24	26
119	1133-0686	617	246	127	54	12	10	11	7	8	9	10	12	9	10	10
120	1539-1604	1075	286	194	183	13	13	12	10	11	13	14	16	13	13	13
121	1434-6028	7722	1849	1420	72	27	21	21	20	22	25	29	32	23	27	30
122	0304-4149	2652	791	577	44	15	15	15	13	15	17	19	21	16	18	19
123	0143-2087	1089	228	155	152	15	14	14	11	12	14	16	17	14	14	14
124	0323-3847	1221	327	230	129	15	13	13	10	12	13	15	17	13	14	14
125	0266-4666	1295	303	208	33	17	14	15	11	12	14	16	18	14	15	15
126	0925-5001	3452	849	611	61	22	18	19	15	17	19	22	24	19	21	22
127	1085-7117	682	183	129	49	13	12	12	9	10	11	12	14	11	11	11
128	0927-5398	1505	358	250	53	18	15	16	12	13	15	17	18	15	16	16
129	0899-8256	2942	696	512	76	20	17	18	15	16	19	21	23	18	20	21
130	0035-9254	1023	212	169	54	14	14	14	11	12	14	15	17	14	14	14
131	0893-9659	9519	1631	1295	95	35	26	27	24	27	31	34	38	29	33	35
132	0926-6003	2408	508	394	78	20	18	18	14	16	18	20	23	18	19	19
133	1368-4221	533	116	86	49	9	12	11	8	9	11	12	13	11	11	10
134	1386-1999	534	120	83	30	13	12	12	8	9	11	12	13	11	11	10
135	0254-5330	4505	1241	824	190	21	18	19	16	18	20	23	25	19	22	24
136	1180-4009	1611	325	236	52	18	16	17	13	14	16	18	20	16	17	16
137	0167-9473	7203	1541	1235	162	26	22	22	20	23	26	29	32	24	28	30
138	0013-1644	1350	262	214	78	16	16	15	12	13	15	17	19	16	16	15
139	1050-5164	2089	373	322	30	20	18	18	14	16	18	20	23	18	19	19
140	1544-6115	1073	260	199	56	15	14	13	10	11	13	15	16	13	14	13
141	1055-6788	1243	314	220	285	12	14	12	11	12	14	15	17	14	14	14
142	1076-9986	655	148	110	60	11	12	12	9	10	11	13	14	12	12	11
143	0025-5718	3127	595	488	60	22	20	20	16	18	20	23	25	20	22	22
144	0036-1410	3275	618	514	85	21	20	20	16	18	21	23	26	21	22	22
145	0740-817X	1881	382	302	44	18	17	17	13	15	17	19	21	17	18	18
146	0167-6687	2779	572	469	37	19	18	18	15	17	19	21	24	19	20	21
147	0364-765X	1237	227	180	61	17	16	16	12	13	15	17	19	15	16	15
148	1017-0405	2048	426	308	190	19	17	17	14	15	17	19	21	17	18	18
149	1369-183X	2904	469	398	90	24	21	20	17	18	21	24	26	21	22	22
150	1545-5963	3954	658	524	72	26	22	23	18	20	23	26	29	23	25	25
151	1064-1246	1887	813	504	40	16	12	13	10	11	13	15	16	12	14	15
152	0025-5564	2637	545	434	61	20	18	18	15	16	19	21	23	19	20	20
153	0036-1399	2359	466	390	63	19	18	18	14	16	18	21	23	18	19	19
154	0022-3239	4134	1005	685	112	24	18	20	16	18	21	23	26	20	22	23
155	0197-9183	1062	195	144	131	15	15	15	11	13	14	16	18	15	15	14
156	0949-2984	777	146	124	25	14	14	13	10	11	13	14	16	13	13	12
157	0178-8051	1744	408	313	47	17	16	16	12	14	16	18	20	16	17	17
158	1435-9871	1565	347	280	51	15	16	15	12	13	15	17	19	16	16	16
159	0091-1798	2227	408	353	56	20	18	18	14	16	18	21	23	19	19	19
160	0895-5646	742	123	103	43	13	14	14	10	12	13	15	16	13	13	12
161	0266-8920	1994	281	226	98	22	20	20	15	17	19	22	24	20	20	19
162	0363-0129	3796	661	534	112	25	21	22	18	20	22	25	28	22	24	24
163	0144-686X	1902	376	287	50	17	17	18	13	15	17	19	21	17	18	18
164	1061-8600	1661	290	237	73	18	17	17	13	15	17	19	21	17	18	17
165	1066-5277	3165	491	380	273	25	22	21	17	19	22	25	27	22	23	23
166	0020-7721	5586	1031	815	180	25	23	23	20	22	25	28	31	24	27	28
167	0303-8300	5093	1260	850	124	25	19	21	17	19	22	25	27	21	24	25
168	0006-341X	3854	717	565	75	24	21	21	17	19	22	25	27	22	24	24
169	0960-1627	854	189	149	36	14	13	13	10	11	13	14	16	13	13	12
170	0305-9049	886	209	157	56	12	13	13	10	11	12	14	16	13	13	12
171	0167-8655	12,864	1417	1249	1129	40	35	33	31	34	39	44	49	38	42	43
172	1932-8184	3207	648	414	74	24	19	22	16	18	20	23	25	20	22	22
173	1613-9372	832	171	134	36	13	14	14	10	11	13	14	16	13	13	12
174	1479-8409	461	115	74	46	11	11	11	8	9	10	11	12	10	10	9
175	1874-8961	1560	275	206	73	19	17	18	13	14	17	19	21	17	17	17
176	0960-3174	1891	408	284	109	19	16	17	13	14	16	19	21	17	18	17
177	1742-5468	3572	1564	950	41	19	13	14	13	14	16	18	20	14	17	20
178	0885-064X	1081	185	149	96	14	16	15	12	13	15	17	18	15	15	14
179	0007-1102	907	149	115	123	14	15	14	11	12	14	16	18	14	14	13
180	0171-6468	1499	215	165	82	17	18	19	14	15	17	20	22	18	18	17
181	1944-0391	484	201	81	28	11	9	11	7	7	8	9	11	9	9	9
182	1726-2135	1007	115	112	66	16	17	16	13	14	17	19	21	17	16	14
183	1544-8444	1703	242	210	56	17	19	19	14	16	18	21	23	19	19	18
184	0032-4728	558	101	87	34	11	13	12	9	10	12	13	15	12	11	11
185	0022-4065	752	113	88	34	14	15	15	11	12	14	15	17	14	13	12
186	0039-3665	913	158	119	176	13	15	13	11	12	14	16	17	14	14	13
187	0168-6577	536	93	80	53	12	13	12	9	10	12	13	15	12	11	10
188	0886-9383	2339	365	286	128	22	20	20	16	17	20	22	25	20	21	20
189	0018-9529	4175	469	387	94	29	27	28	21	23	27	30	33	27	28	27
190	1054-1500	5630	936	774	80	27	24	24	20	23	26	29	32	25	28	29
191	0304-4076	5332	723	609	165	30	26	26	21	24	27	31	34	27	29	29
192	0006-3444	2406	392	314	85	22	20	20	15	17	20	22	25	20	21	20
193	0964-1998	1287	234	177	50	17	16	16	12	13	15	17	19	16	16	15
194	1932-6157	2740	524	373	102	22	19	20	15	17	19	22	24	19	21	21
195	1468-1218	12,517	1271	1139	238	42	37	36	31	35	40	45	50	39	43	43
196	0025-5610	3997	567	442	194	27	24	24	19	21	24	27	30	25	26	26
197	1436-3240	3874	661	562	66	24	22	21	18	20	23	25	28	23	24	24
198	0167-6911	7259	731	617	351	37	32	32	26	29	33	37	42	34	35	35
199	0305-0548	13,373	1261	1135	156	45	39	39	33	37	42	47	52	42	45	45
200	0040-1706	1141	235	153	79	16	15	16	11	12	14	16	18	14	15	14
201	0165-0114	7962	1106	818	108	33	28	31	24	27	31	35	39	30	33	34
202	0883-7252	2055	286	234	108	22	20	20	15	17	20	22	25	20	20	19
203	0272-4332	6416	871	687	86	33	27	29	23	25	29	33	36	29	31	31
204	0277-6715	10,506	1780	1314	623	35	27	28	25	28	32	36	40	30	34	37
205	1568-4539	976	119	106	109	15	17	16	13	14	16	18	20	16	15	14
206	0022-2496	1417	199	160	82	19	18	18	14	15	17	19	22	18	18	16
207	0033-3123	1431	231	172	288	14	17	16	13	14	17	19	21	17	17	16
208	0951-8320	9529	926	850	95	37	35	35	29	32	37	42	46	37	39	39
209	0304-3800	13,918	1689	1511	412	36	34	33	31	34	39	44	49	38	42	44
210	1384-5810	2334	238	198	137	24	24	24	18	20	23	26	28	23	23	21
211	0169-7439	5880	726	645	187	30	28	27	23	25	29	33	36	29	31	31
212	1538-6341	1341	264	132	147	17	16	18	12	13	15	17	19	16	16	15
213	0030-364X	5098	554	487	120	30	29	29	23	25	29	32	36	29	30	30
214	0098-7921	1855	198	153	143	22	22	22	16	18	21	23	26	21	21	19
215	1465-4644	2347	304	253	142	23	22	21	17	18	21	24	26	22	22	21
216	0199-0039	1110	140	108	95	16	18	17	13	14	17	19	21	17	16	15
217	1052-6234	4321	414	345	765	25	29	26	22	25	28	32	36	29	29	28
218	0735-0015	1932	245	186	258	22	21	20	16	17	20	22	25	20	20	19
219	0167-9236	10,594	923	797	458	42	38	38	31	35	40	45	50	40	42	42
220	0162-1459	5231	663	519	156	31	27	28	22	24	28	31	35	28	29	29
221	0049-1241	803	115	99	148	14	15	13	11	12	14	16	18	14	14	13
222	0378-8733	2879	231	214	391	22	28	25	21	23	26	30	33	27	26	24
223	1470-160X	16,653	1636	1516	214	44	40	39	35	39	44	50	55	43	48	49
224	0070-3370	3714	420	376	74	26	26	26	20	22	26	29	32	26	27	26
225	0962-2802	1476	211	153	102	21	18	19	14	15	17	20	22	18	18	17
226	0090-5364	5835	486	433	315	31	33	33	26	29	33	37	41	34	34	33
227	0027-3171	1886	196	151	460	18	22	19	17	18	21	24	26	21	21	19
228	0883-4237	1909	237	151	375	21	21	20	16	17	20	22	25	20	20	19
229	1532-4435	14,005	1121	841	966	55	42	45	35	39	45	50	56	45	48	47
230	1369-7412	3186	169	149	475	23	32	30	25	27	31	35	39	31	29	26
231	1070-5511	1374	187	152	94	18	18	18	14	15	17	19	22	18	17	16

*C* the total number of citations, *T* the total number of papers, *T*_1_ total number of papers cited at least once, *C*_1_ the total number of citations of the most cited paper, *h* the actual value of the *h*-index; $h_{W}^{\left( 0 \right)}$, $\tilde{h}_{W}^{\left( 1 \right)}$ Lambert-*W* formulas for the *h*-index, $h_{\text{SG}} \left( {\gamma_{0} } \right)$ the Glänzel–Schubert formula, for different values of *γ*_0_, *γ*_0_ = 0.63, 0.7, 0.8, 0.9, 1, $h_{\text{BS}} \left( {q_{0} } \right)$ the numerical solution of Eq. (18), for different values of *q*_0_, *q*_0_ = 1.2, 1.4, 1.6

### A second dataset of journals

#### Journal selection

We also analyzed a second dataset, based on the citation data of the top 100 journals, within the Scopus subject area of “Economics, Econometrics and Finance”, ranked according to the Scopus journal impact factor, i.e. the Impact per Publication (IPP) 2014. The list (let us call it the EE&F list) may be found at http://www.journalindicators.com and it consists of journals with a minimum number of 50 publications. We recall that the IPP 2014 of a journal is basically the average number of citations received by papers published in 2014 (registered in the Scopus database), to papers published by the same journal from 2011 until 2013. In particular, Scopus takes account of the following types of citable items and citing sources: articles, reviews, and conference papers. All other documents (e.g. notes, letters, articles in press, erratum, etc.) are excluded from the computation. We downloaded from Scopus the citation data of all 100 journals on the aforementioned list during the last week of April, 2016. The dataset obtained included 19,889 publications receiving a total of 74,096 citations (during 2014). The complete list of these journals is reported in Table 3, where each journal is identified by its ISSN code. Differently from above, we excluded all non-citable items (e.g. notes, etc.) in order to obtain sets of publications as close as possible to those employed for the computation of IPPs by Scopus. Once the set of papers for each journal has been selected, it is possible to request a citation report (“view citation overview”) and download the citations per paper received in the year 2014: that is, all and only the citations needed for the computation of the IPP 2014. In fact, we found some positive differences between the actual values of $m = C/T$, with an average value over all 100 journals of 3.8, and the official IPPs 2014, with an average value of 3. These differences may be due to: (1) a delayed update of the database (the IPPs were published by Scopus in June 2015), and (2) a larger set of citing sources and documents (with Scopus, it is not possible to limit the citation report to particular citing sources or documents). Similar differences between official and observed values have been found and discussed, for instance, by Leydesdorff and Opthof (2010), Stern (2013) and Seiler and Wohlrabe (2014). Nonetheless, in this case the ACPP $m = C/T$ should, theoretically, represent a 3-year synchronous impact factor for the year 2014 (Ingwersen et al. 2001; Ingwersen 2012) in that we considered only citations received during 2014 of papers published within the previous 3 years. For each journal, we manually computed the actual value *h* of the *h*-index as the largest number of papers published in the journal between 2011 and 2013 and which obtained at least *h* citations each in the year 2014. Ultimately, we obtained a synchronous *h*-index (Bar-Ilan 2010), for a 1-year citation window.

**Table 3:** Basic statistics for the EE&F list of journals and the approximations of the Hirsch *h*-index calculated by means of different formulas (rounded values)

#	ISSN code	*C*	*T*	*T*_1_	$C_{\text{1}}$	*h*	$h_{W}^{\left( 0 \right)}$	$\tilde{h}_{W}^{\left( 1 \right)}$	$h_{\text{SG}} \;\left( {.63} \right)$	$h_{\text{SG}} \;\left( {.7} \right)$	$h_{\text{SG}} \;\left( {.8} \right)$	$h_{\text{SG}} \;\left( {.9} \right)$	$h_{\text{SG}} \;\left( 1 \right)$	$h_{\text{BS}} \;\left( {1.2} \right)$	$h_{\text{BS}} \left( {1.4} \right)$	$h_{\text{BS}} \;\left( {1.6} \right)$
1	0022-0515	697	69	63	61	15	16	15	12	13	15	17	19	15	14	12
2	1531-4650	1161	127	117	58	18	19	18	14	15	18	20	22	18	17	15
3	1557-1211	1773	193	173	119	21	21	20	16	18	20	23	25	21	20	19
4	1540-6261	1529	190	178	54	17	19	19	15	16	18	21	23	19	19	17
5	0895-3309	995	133	111	44	15	17	16	12	14	16	18	20	16	15	14
6	1547-7185	1196	153	143	41	17	18	17	13	15	17	19	21	17	17	15
7	0092-0703	1015	140	128	111	15	17	15	12	14	16	18	19	16	15	14
8	0304-405X	2413	412	372	48	20	19	19	15	17	19	22	24	20	20	20
9	1468-0262	1014	187	171	35	14	15	14	11	12	14	16	18	14	14	14
10	1523-2409	434	81	71	26	10	11	11	8	9	11	12	13	11	10	9
11	1537-534X	483	92	79	56	10	12	11	9	10	11	12	14	11	11	10
12	1465-7368	1389	288	256	38	16	16	15	12	13	15	17	19	15	16	15
13	1540-6520	1062	175	147	52	15	16	15	12	13	15	17	19	15	15	14
14	1478-6990	795	155	140	38	13	14	13	10	11	13	14	16	13	13	12
15	1945-7790	516	113	103	22	10	12	11	8	9	11	12	13	11	11	10
16	0002-8282	3303	723	562	48	21	19	19	16	17	20	22	25	19	21	22
17	1945-7715	422	91	78	38	9	11	10	8	9	10	11	13	10	10	9
18	1741-6248	361	55	52	52	10	11	10	8	9	11	12	13	10	10	9
19	1469-5758	272	65	46	26	10	9	9	7	7	8	9	10	8	8	7
20	0165-4101	517	118	99	22	11	11	11	8	9	11	12	13	11	11	10
21	0925-5273	4678	1036	888	92	22	20	19	17	19	22	25	28	21	24	25
22	1542-4774	641	148	122	74	10	12	11	9	10	11	13	14	12	12	11
23	1537-5277	1086	234	213	24	12	14	13	11	12	14	15	17	14	14	14
24	0921-3449	1723	421	363	33	15	15	14	12	13	15	17	19	15	16	16
25	1467-937X	688	192	147	32	11	11	11	9	9	11	12	14	11	11	11
26	1945-774X	422	109	93	49	8	10	9	7	8	9	11	12	10	10	9
27	1873-6181	2683	667	565	26	16	17	16	14	15	18	20	22	17	19	20
28	1547-7193	948	213	188	56	13	14	12	10	11	13	15	16	13	13	13
29	1086-4415	324	57	49	36	10	10	10	8	9	10	11	12	10	9	8
30	1741-2900	234	54	42	34	8	9	8	6	7	8	9	10	8	8	7
31	1530-9142	1065	292	241	27	13	13	12	10	11	13	14	16	13	13	13
32	1530-9290	887	242	208	38	11	12	11	9	10	12	13	15	12	12	12
33	0001-4826	837	217	178	48	12	12	12	9	10	12	13	15	12	12	12
34	1090-9516	639	154	134	23	12	12	11	9	10	11	12	14	11	11	11
35	1547-7215	239	60	54	14	8	9	8	6	7	8	9	10	8	8	7
36	1941-1383	246	66	51	33	8	9	8	6	7	8	9	10	8	8	7
37	0921-8009	2620	675	567	34	17	16	16	14	15	17	19	22	17	19	19
38	0024-6301	248	58	44	33	9	9	8	6	7	8	9	10	8	8	7
39	1468-2710	586	142	122	36	10	12	11	8	9	11	12	13	11	11	10
40	1468-0297	760	210	179	29	10	12	11	9	10	11	13	14	11	12	11
41	1066-2243	355	85	73	27	9	10	9	7	8	9	10	11	9	9	8
42	1475-679X	398	111	86	21	10	10	10	7	8	9	10	11	9	9	9
43	0308-597X	1557	475	399	35	12	13	12	11	12	14	15	17	14	15	15
44	0022-1996	794	247	191	22	11	11	11	9	10	11	12	14	11	12	11
45	1096-0449	673	183	142	25	11	12	11	9	9	11	12	14	11	11	11
46	1573-6938	340	99	72	68	7	9	8	7	7	8	9	11	9	9	8
47	2041-417X	178	55	35	26	7	7	7	5	6	7	7	8	7	7	6
48	0306-9192	951	291	224	35	14	12	12	9	10	12	13	15	12	12	12
49	1537-2707	422	139	86	73	9	9	9	7	8	9	10	11	9	9	9
50	0013-0095	175	51	39	26	8	7	7	5	6	7	8	8	7	7	6
51	1052-150X	265	70	57	17	8	9	8	6	7	8	9	10	8	8	7
52	1533-4465	179	56	28	25	8	7	8	5	6	7	7	8	7	7	6
53	1526-548X	634	182	142	61	11	11	10	8	9	10	12	13	11	11	11
54	1873-5991	1725	540	426	22	13	14	13	11	12	14	16	18	14	15	16
55	1389-5753	231	64	56	17	8	8	8	6	7	8	8	9	8	7	7
56	1572-3089	268	86	71	24	7	8	8	6	7	8	8	9	8	8	7
57	1468-1218	2068	716	522	35	14	13	13	11	13	15	16	18	14	16	17
58	0304-3878	876	295	220	35	13	11	11	9	10	11	12	14	11	12	12
59	0047-2727	959	331	246	74	11	11	11	9	10	11	13	14	11	12	12
60	0969-5931	652	213	172	16	9	11	10	8	9	10	11	13	10	11	10
61	1532-8007	270	102	78	23	7	8	7	6	6	7	8	9	7	7	7
62	1075-4253	245	80	69	10	7	8	7	6	6	7	8	9	7	7	7
63	1386-4181	192	68	47	24	7	7	7	5	6	7	7	8	7	7	6
64	0265-1335	252	82	62	12	8	8	8	6	6	7	8	9	8	7	7
65	1537-5307	214	79	61	11	7	7	7	5	6	7	8	8	7	7	6
66	0301-4207	490	165	122	30	9	10	9	7	8	9	10	11	9	9	9
67	1096-1224	200	61	57	22	7	8	7	5	6	7	8	9	7	7	6
68	1467-6419	349	121	90	18	9	9	8	6	7	8	9	10	8	8	8
69	1932-443X	163	53	47	11	6	7	6	5	6	6	7	8	6	6	6
70	1756-6916	433	167	125	19	9	9	9	7	7	8	9	10	8	9	9
71	0304-3932	389	154	105	45	8	9	8	6	7	8	9	10	8	8	8
72	1572-3097	265	107	78	14	7	8	7	5	6	7	8	9	7	7	7
73	1464-5114	358	119	106	19	7	9	8	6	7	8	9	10	8	8	8
74	1911-3846	437	156	110	31	10	9	9	7	7	9	10	11	9	9	9
75	1096-0473	220	87	62	17	7	7	7	5	6	7	7	8	7	7	6
76	1095-9068	325	126	99	13	8	8	8	6	7	8	8	9	8	8	8
77	1389-9341	817	325	252	17	10	10	10	8	9	10	11	13	10	11	11
78	0217-4561	402	148	123	13	8	9	8	6	7	8	9	10	8	9	8
79	1548-8004	238	101	77	8	7	7	7	5	6	7	7	8	7	7	7
80	0304-4076	1037	404	305	28	12	11	10	9	10	11	12	14	11	12	12
81	0038-0121	218	74	49	38	7	8	7	5	6	7	8	9	7	7	6
82	0928-7655	340	133	93	38	8	8	8	6	7	8	9	10	8	8	8
83	1747-762X	205	91	60	38	6	7	6	5	5	6	7	8	6	6	6
84	1566-0141	273	110	87	16	7	8	7	6	6	7	8	9	7	7	7
85	1392-8619	368	117	79	45	9	9	9	7	7	8	9	10	9	9	8
86	1573-0913	719	261	198	18	11	10	10	8	9	10	11	13	10	11	11
87	1475-1461	244	83	64	26	8	8	7	6	6	7	8	9	7	7	7
88	1099-1255	372	163	113	15	8	8	8	6	7	8	9	9	8	8	8
89	0176-2680	416	179	135	18	7	9	8	6	7	8	9	10	8	8	8
90	1096-6099	242	113	78	25	6	7	7	5	6	6	7	8	7	7	6
91	1432-1122	175	89	64	8	5	6	6	4	5	6	6	7	6	6	6
92	0929-1199	553	244	172	28	8	9	9	7	8	9	10	11	9	9	9
93	1573-0697	2627	934	717	29	13	14	13	12	14	16	18	19	15	17	18
94	1467-0895	159	57	44	10	6	7	7	5	5	6	7	8	6	6	6
95	0378-4266	1993	893	621	36	13	12	11	10	12	13	15	16	12	14	15
96	1877-8585	167	64	50	15	6	7	6	5	5	6	7	8	6	6	6
97	1179-1896	272	127	88	9	6	7	7	5	6	7	8	8	7	7	7
98	0308-5147	231	88	60	14	8	8	8	5	6	7	8	8	7	7	7
99	1043-951X	449	194	145	19	8	9	8	6	7	8	9	10	8	9	9
100	0168-7034	176	74	41	13	8	7	7	5	5	6	7	7	6	6	6

*C* the total number of citations, *T* the total number of papers, *T*_1_ the total number of papers cited at least once, *C*_1_ the total number of citations of the most cited paper, *h* the actual value of the *h*-index, $h_{W}^{\left( 0 \right)}$, $\tilde{h}_{W}^{\left( 1 \right)}$ Lambert-*W* formulas for the *h*-index, $h_{\text{SG}} \left( {\gamma_{0} } \right)$ Glänzel–Schubert formula, for different values of *γ*_0_, *γ*_0_ = 0.63, 0.7, 0.8, 0.9, 1; $h_{\text{BS}} \left( {q_{0} } \right)$ the numerical solution of Eq. (18), for different values of *q*_0_, *q*_0_ = 1.2, 1.4, 1.6

#### Estimating the *h*-index

In the same way as above, for each journal we manually computed the actual value *h* of the *h*-index. Table 3 reports, for each journal, the *h*-index, *h*, and the other indicators also considered in Table 2, namely $h_{W}^{\left( 0 \right)}$, $\tilde{h}_{W}^{\left( 1 \right)}$, $h_{\text{SG}} \left( {\gamma_{0} } \right)$, for $\gamma_{0} = 0.63, 0.7, 0.8, 0.9, 1$, the numerical solution $h_{\text{T}} \left( {q_{0} } \right)$ of Eq. (18), for different values of *q*_0_, namely $q_{0} = 1.2, 1.4, 1.6$, as well as the simple basic metrics *C*, *T*, *T*_1_ and *C*_1_.

## Discussion and conclusion

The *h*-index is, today, one of the tools most commonly used to rank journals (Braun et al. 2006; Vanclay 2007, 2008; Schubert and Glänzel 2007; Bornmann et al. 2009; Harzing and van der Wal 2009; Liu et al. 2009; Hodge and Lacasse 2010; Bornmann et al. 2012; Mingers et al. 2012; Xu et al. 2015). Indeed, its value is currently provided by all the three major citation databases, WoS, Scopus and GS. In an earlier study (Bertoli-Barsotti and Lando 2015) the Lambert-*W* formula for the *h*-index $\tilde{h}_{W}^{\left( 1 \right)}$ was proved to be a good estimator of the *h*-index for authors. In this paper, we have extended the empirical study to the case of the *h*-index for journals. One of the major differences between the case of an individual scientist and that of a journal, for the computation of the *h*-index, is the role played by publication and citation time windows, and the approach adopted for the analysis and interpretation of the citation process (“prospective” vs “retrospective”; Glänzel 2004). As stressed by Braun et al. (2006): “The journal *h*-index would not be calculated for a “life-time contribution”, as suggested by Hirsch for individual scientists, but for a definite period”. In fact, “Hirsch did not limit the period in which the citations were received” (Bar-Ilan 2010). Unlike the case of individual scientists, and in view of a comparative assessment, calculations of a journal’s *h*-index must be timed (note that a notion of “timed *h*-index” has also been recently introduced by Schreiber (2015), for the case of individual scientists), i.e. it must be referred to standardized time periods of journal coverage, for example of 2, 3 or 5 years, as is usually done for the computation of the impact factor, in order to limit the typical size-dependency of the *h*-index—that is, its dependency on the total number of publications (an indicator is said to be size-dependent if it never decreases when new publications are added, Waltman 2016). A journal’s “impact factor” is essentially a time-limited version of the average number of citations by papers published in the journal in a given period of time. Several types of “impact factors” may be defined, depending on different time windows considered for publication and citation data and, possibly, different approaches to the analysis of the citation process, leading to synchronous or diachronous impact factors (Ingwersen et al. 2001; Ingwersen 2012). In its WoS form, the publication window is 2 years (defining the 2-year Impact Factor, IF) or 5 years (defining the 5-year Impact Factor, IF5), while Scopus adopts a 3-year publication window for its IPP. In all these cases, the impact factor is computed in a synchronous mode, i.e. the citations used for the calculation are all received during the same fixed period—1 year, in these cases.

In this paper, we first presented the Lambert-*W* formula for the *h*-index in two versions (differing on the basis of the various citation metrics on which they depend), a basic version and an improved version, respectively $h_{W}^{\left( 0 \right)}$ and $\tilde{h}_{W}^{\left( 1 \right)}$. Then we tested, by means of an empirical study, their efficiency and effectiveness, as well as:

1. that of another popular theoretical model for the *h*-index that has been successfully applied elsewhere to the same type of application, i.e. the Glänzel–Schubert formula, $h_{\text{SG}} \left( {\gamma_{0} } \right)$, for different values of the free parameter *γ*_0_, and secondly,
2. that given by the numerical solution $h_{\text{BS}} \left( {q_{0} } \right)$ of Eq. (18), for different values of the free parameter *q*_0_.

We compared the performances of these formulas as estimators of the *h*-index—in particular, in terms of accuracy and robustness—with an empirical study conducted on two different samples of journals. We computed the *h*-index manually, on the basis of citations downloaded. In our empirical study, in the first dataset (S&MM), the ACPP $m = C/T$ can be interpreted as a diachronous impact factor (Ingwersen et al. 2001; Ingwersen 2012), because for each paper the citations are counted from the moment of publication until the time of accessing the database (as in the case of individual scientists). More specifically, we computed an “impact factor” involving a 6-year citation window over a 5-year publication window. As to be expected, due to the larger citation window, we obtained, for all 231 journals, the averages of 4.4 and 1.5 respectively for *m* and *IF*5{2014}, the traditional 5-year impact factors 2014, as published by WoS in its Journal Citation Report. Moreover, we also observed a high level of Pearson correlation, *ρ*, between *m* and *IF*5{2014}, that is: $\rho \left( {m,IF5\left\{ {2014} \right\}} \right) = 0.87$ (quite similar to that observed between *IF*5{2014} and *IF*{2014}, the WoS 2-year and impact factors 2014, that is: $\rho \left( {IF\left\{ {2014} \right\},IF5\left\{ {2014} \right\}} \right) = 0.90$). Instead, in the second dataset (EE&F), *m* can be interpreted as a 3-year impact factor in its ordinary synchronous version, as computed by Scopus. Hence, following the terminology of Bar-Ilan (2010, 2012), we obtained a diachronous and a synchronous *h*-index, respectively, in the first and second empirical study. To evaluate the measure of fit of an estimate of the *h*-index, say $\hat{h}_{j}$ (rounded to the nearest natural number), with respect to the exact value *h*_*j*_, we computed the absolute relative error ${\text{ARE}}_{j} = \left| {{{\left( {\hat{h}_{j} - h_{j} } \right)} \mathord{\left/ {\vphantom {{\left( {\hat{h}_{j} - h_{j} } \right)} {h_{j} }}} \right. \kern-0pt} {h_{j} }}} \right|$ and the squared relative error ${\text{SRE}}_{j} = \left( {{{\left( {\hat{h}_{j} - h_{j} } \right)} \mathord{\left/ {\vphantom {{\left( {\hat{h}_{j} - h_{j} } \right)} {h_{j} }}} \right. \kern-0pt} {h_{j} }}} \right)^{2}$ for each journal *j*, *j* = 1,…,*J*. Then, as a criterion with which to assess the overall quality of the various estimators considered in the paper, we computed the mean absolute relative error, ${\text{MARE}}\left( {\hat{h}} \right) = \sum\nolimits_{j = 1}^{J} {{{{\text{ARE}}_{j} } \mathord{\left/ {\vphantom {{{\text{ARE}}_{j} } J}} \right. \kern-0pt} J}}$ and the root mean squared relative error ${\text{RMSRE}}\left( {\hat{h}} \right) = \sqrt {\sum\nolimits_{j = 1}^{J} {{{{\text{SRE}}_{j} } \mathord{\left/ {\vphantom {{{\text{SRE}}_{j} } J}} \right. \kern-0pt} J}} }$, for each estimator.

**Fig. 1:**
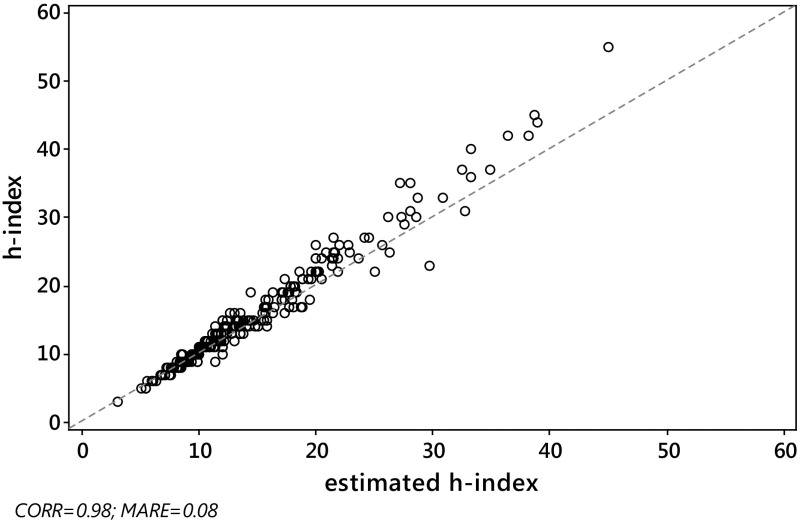
S&MM dataset: scatterplot of *h* versus $\tilde{h}_{W}^{\left( 1 \right)}$. Pearson correlation $\rho \left( {h,\tilde{h}_{W}^{\left( 1 \right)} } \right) = 0.98$, ${\text{MARE}}\left( {\tilde{h}_{W}^{\left( 1 \right)} } \right) = 0.08$. The *dashed line* is identity, so ideally all the points should overlie this line

**Fig. 2:**
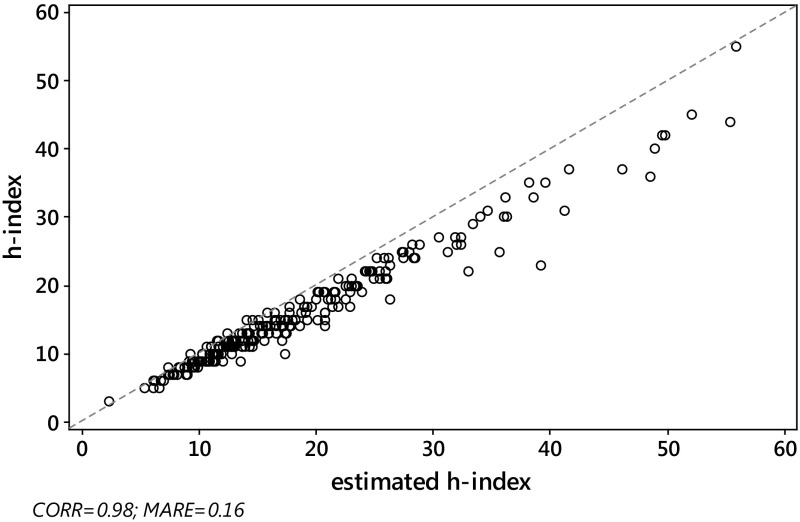
S&MM dataset: scatterplot of *h* vs Glänzel–Schubert formula $h_{\text{SG}} \left( 1 \right)$. Pearson correlation $\rho \left( {h,h_{\text{SG}} \left( 1 \right)} \right) = 0.98$, ${\text{MARE}}\left( {h_{\text{SG}} \left( 1 \right)} \right) = 0.16$. The *dashed line* is identity, so ideally all the points should overlie this line

**Fig. 3:**
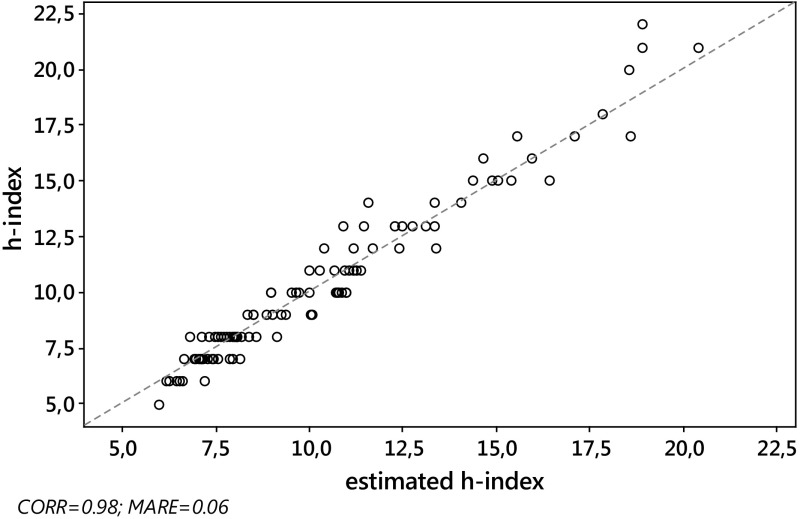
EE&F dataset. Scatterplot of *h* versus $\tilde{h}_{W}^{\left( 1 \right)}$. Pearson correlation $\rho \left( {h,\tilde{h}_{W}^{\left( 1 \right)} } \right) = 0.98$, ${\text{MARE}}\left( {\tilde{h}_{W}^{\left( 1 \right)} } \right) = 0.05$. The *dashed line* is identity, so ideally all the points should overlie this line

**Fig. 4:**
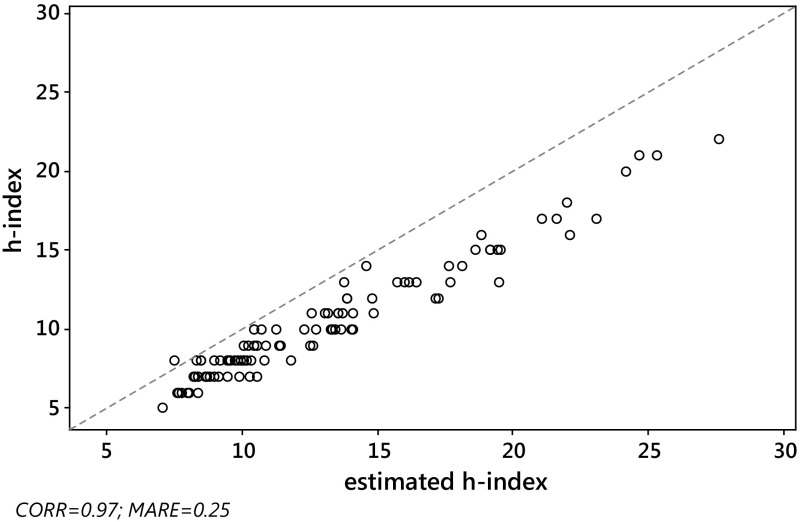
EE&F dataset: versus Glänzel–Schubert formula $h_{\text{SG}} \left( 1 \right)$. Pearson correlation $\rho \left( {h,h_{\text{SG}} \left( 1 \right)} \right) = 0.97$, ${\text{MARE}}\left( {h_{\text{SG}} \left( 1 \right)} \right) = 0.25$. The *dashed line* is identity, so ideally all the points should overlie this line

1. As expected, the Pearson correlation between the actual value *h* of the *h*-index and each of its estimates $h_{W}^{\left( 0 \right)}$, $\tilde{h}_{W}^{\left( 1 \right)}$ and $h_{\text{SG}} \left( {\gamma_{0} } \right)$, was very high, for both S&MM and EE&F datasets. In particular, this confirms previous empirical results concerning the formula $h_{\text{SG}}$ (see Schubert and Glänzel 2007; Glänzel 2007). Indeed, *ρ* always exceeded 0.97. More specifically, we found the following: for the S&MM dataset, $\rho ( {h,h_{W}^{( 0 )} }) = 0.97$ and $\rho ( {h,\tilde{h}_{W}^{( 1 )} } ) = \rho ( {h,h_{\text{SG}} } ) = 0.98$; for the EE&F dataset,$\rho ( {h,h_{W}^{( 0 )} } ) = \rho ( {h,h_{\text{SG}} } ) = 0.97$ and $\rho ( {h,\tilde{h}_{W}^{( 1 )} } ) = 0.98$. Nevertheless, as can be seen from Figs. 2 and 4, a high correlation does not specifically identify a “good” estimator for the *h*-index. Formula $\tilde{h}_{W}^{( 1 )}$ yielded similar levels of correlation, but a much lower level of MARE, see Figs. 1 and 3 (be aware that the figures refer to non-rounded values of the estimates). Note that the correlation between the *h*-index and $h_{\text{SG}} \left( {\gamma_{0} } \right)$ does not depend on the unknown value of $\gamma_{0}$, while, at the same time, the MARE of $h_{SG} \left( {\gamma_{0} } \right)$ depends heavily on the choice of $\gamma_{0}$. As can be seen from Table 4, at its best (among the values of $\gamma_{0}$ tested), the error of $h_{SG} \left( {\gamma_{0} } \right)$ reached its minimum (in terms of both MARE and RMSRE), for $\gamma_{0} = 0.9$, for the dataset S&MM, while for the EE&F dataset the error of $h_{SG} \left( {\gamma_{0} } \right)$ is at its minimum for a slightly different value of *γ*_0_, i.e. *γ*_0_ = 0.8. This confirms that, for fixed values of *γ*_0_, the effectiveness of the formula may depend on the length of the citation window considered (Glänzel 2008) and, finally, that there is no “universal” optimal value for the constant *γ*_0_ in the formula $h_{\text{SG}} \left( {\gamma_{0} } \right)$. Instead, for both datasets, the formula $\tilde{h}_{W}^{\left( 1 \right)}$ gives similar, and even smaller, levels of error (in terms of both MARE and RMSRE).

**Table 4:** Relative accuracy, computed in terms of MARE and RMSRE (in italic), of different estimators of the *h*-index. For each dataset, the smallest error is indicated by a boldface number

Journal dataset	MARE RMSRE $h_{W}^{\left( 0 \right)}$	MARE RMSRE $\tilde{h}_{W}^{\left( 1 \right)}$	MARE RMSRE $h_{\text{SG}} \;\left( {.63} \right)$	MARE RMSRE $h_{\text{SG}} \;\left( {.7} \right)$	MARE RMSRE $h_{\text{SG}} \;\left( {.8} \right)$	MARE RMSRE $h_{\text{SG}} \;\left( {.9} \right)$	MARE RMSRE $h_{\text{SG}} \;\left( 1 \right)$	MARE RMSRE $h_{\text{BS}} \;\left( {1.2} \right)$	MARE RMSRE $h_{\text{BS}} \;\left( {1.4} \right)$	MARE RMSRE $h_{\text{BS}} \;\left( {1.6} \right)$
S&MM	0.104	0.076	0.272	0.193	0.099	0.076	0.163	0.103	**0.065**	0.076
	* 0.133*	*0.100*	*0.283*	*0.207*	*0.122*	*0.117*	*0.198*	*0.129*	***0.094***	*0.103*
EE&F	0.092	**0.050**	0.217	0.127	0.058	0.130	0.251	0.058	0.072	0.092
	*0.120*	***0.079***	*0.229*	*0.149*	*0.088*	*0.158*	*0.275*	*0.093*	*0.108*	*0.124*
2. The approach that consists of obtaining the numerical solution $h_{\text{BS}} \left( {q_{0} } \right)$ of Eq. (18) was also considered. We tentatively tested this method for nine different values of the free parameter *q* between 1 and 2, i.e. *q*_0_ = 1.1, 1.2,…,1.9. As expected, the resulting estimates were more or less accurate depending on the set value of *q*_0_. Of the nine values of *q*_0_ tested, the smallest estimation error was obtained for a *q*_0_ value equal to around 1.4 (MARE = 0.065; RMSRE = 0.094), for the S&MM dataset, and for a *q*_0_ value equal to around 1.2 (MARE = 0.058; RMSRE = 0.093) for the EE&F dataset (see Table 4). Ultimately, *h*_T_ was found to be the most accurate estimator (if one takes *q*_0_ = 1.4), of those included in Table 4, for the S&MM dataset and the third best (if one takes *q*_0_ = 1.2), for the EE&F dataset. Overall, the errors are not dramatically different in the range of *q* between 1.2 and 1.6, and then a value of *q*_0_ = 1.5, also tested by Bletsas and Sahalos (2009), may be a good compromise solution. The Pearson correlation between the actual value *h* of the *h*-index and its estimate $h_{\text{BS}} \left( {q_{0} } \right)$ varies slightly according to the selected value of *q*_0_, but it is still very high: in particular, for *q*_0_ = 1.5, we obtain $\rho \left( {h,h_{\text{BS}} \left( {q_{0} } \right)} \right) = 0.98$ for the S&MM dataset and $\rho \left( {h,h_{\text{BS}} \left( {q_{0} } \right)} \right) = 0.96$ for the EE&F dataset. Hence, overall, the method may lead to a very good fit, but it has two main drawbacks. First, the expression of $h_{\text{BS}} \left( {q_{0} } \right)$ is not given by any explicit formula. Second, this method continues to suffer from the problem of the conventional choice of an unknown parameter, in that we do not know a priori the value of the parameter *q* that will yield the “smallest” estimation error.

In conclusion, basically, the same type of equation (see Eqs. 4, 10), describes the relationship between the *h*-index and other simple citation metrics. The Lambert-*W* formula for the *h*-index works well (also) for estimating the *h*-index for journals—especially in its improved version (13). As can be deduced from our empirical study, this still holds true for different scientific areas, for different time windows for publication and citation, for different types of “citable” documents, and for different approaches to the analysis of the citation process (“prospective” vs “retrospective”; Glänzel 2004). At the same time, the Glänzel–Schubert class of models seems to be much less robust and reliable as an estimator of the *h*-index, because its accuracy closely depends on a conventional choice of one or more unknown parameters. We may accordingly conclude that $h_{W}^{\left( 0 \right)}$ and $\tilde{h}_{W}^{\left( 1 \right)}$ are quite effective “universal” (in the sense that they are ready-to-use) informetric functions that work well for estimating the *h*-index, for a sufficiently wide range of values. Indeed, our empirical analysis, though preliminary, suggests that the fit is very good, at least for the datasets that we studied, and for values of its arguments that are not too large, namely, *h* < 40, *T* < 2000 and *m* < 20, which may be considered standard values for the cases of both and scientists journals within time-spans of 2–5 years.
